# PERIPHERAL ARTERIAL DISEASE AND SEDENTARY TIME IN OLDER ADULTS

**DOI:** 10.1093/geroni/igz038.3362

**Published:** 2019-11-08

**Authors:** Dottington Fullwood, Mary McDermott, Todd M Manini

**Affiliations:** 1 University of Florida, Gainesville, Florida, United States; 2 Northwestern University, Chicago, Illinois, United States

## Abstract

Peripheral artery disease (PAD) is associated with increased rates of physical disability in older adults, yet few interventions exist to reduce this risk. Intermittent claudication, exertional calf symptoms that resolve within 10 minutes of rest, is the classic symptom for PAD, but many people with PAD are absent of these symptoms. Ankle brachial index (ABI) is a non-invasive measure that identifies the presence and severity of lower extremity arterial obstruction due to atherosclerosis. We studied whether abnormal ABI is associated with increased time spent in sedentary behavior in a large sample of community-dwelling older men and women (70-89 years) enrolled in the Lifestyle Interventions and Independence for Elders (LIFE) study. Older adults underwent an ABI test and then wore a tri-axial accelerometer on the hip for up to seven days. Total accumulated sedentary time and sedentary time spent in bout lengths of 10minutes or more, 30 minutes or more, and 60 minutes or more were calculated. ABI values, divided into PAD (<.90, n=156) and non-PAD (0.90 - 1.40, n=960), were evaluated in covariate-adjusted regression models adjusting for age, body mass index, comorbidity presence, gender and smoking. Older adults with PAD had significantly higher total accumulated time spent in sedentary behavior than those without PAD (13.1 minutes per day, p<0.02). No associations were found with longer bout lengths of sedentary time. These results suggest that older adults with PAD accumulate more time in shorter bouts of sedentary behavior. Future interventions may consider targeting short sedentary bout-lengths for reducing PAD symptoms.

